# FHL3 Regulates Vascular Smooth Muscle Cell Phenotypic Switching Through the MRTFB-SRF Signaling Axis

**DOI:** 10.3390/cells15161475

**Published:** 2026-08-17

**Authors:** Xiaoxin Huang, Heming Zhang, Yanhong Zhang, Charles U. Solomon, David G. McVey, Shu Ye

**Affiliations:** 1 Division of Basic Medicine, Shantou University Medical College, Shantou 515041, China; 19xxhuang@stu.edu.cn (X.H.); yhzhang1@stu.edu.cn (Y.Z.); 2 Cardiovascular-Metabolic Disease Translational Research Programme, Department of Medicine, Yong Loo Lin School of Medicine, National University of Singapore, Singapore 117599, Singapore; hemingz@nus.edu.sg; 3 Division of Cardiovascular Sciences, University of Leicester, Leicester LE3 9QP, UK; cs806@leicester.ac.uk (C.U.S.); dgm14@leicester.ac.uk (D.G.M.); 4 Leicester British Heart Foundation Centre of Research Excellence, University of Leicester, Leicester LE3 9QP, UK; 5 The First Affiliated Hospital of Shantou University Medical College, Shantou 515041, China

**Keywords:** coronary artery disease, causal gene, VSMC, FHL3, MYH11

## Abstract

**Highlights:**

**What are the main findings?**

- FHL3 is identified as a CAD-associated gene that suppresses the contractile VSMC phenotype and enhances proliferation and migration.
- FHL3 associates with myocardin-related transcription factor-B (MRTFB) and attenuates MRTFB–serum response factor–dependent transcription of contractile marker genes.
- CAD-associated genetic variation influences *FHL3* expression in VSMCs, linking genetic risk to VSMC plasticity.

**What are the implications of the main findings?**

- These findings define a new FHL3-MRTFB signaling axis controlling VSMC plasticity and provide mechanistic insight into how the CAD risk locus modulates vascular cell behavior.
- Targeting this pathway may represent a strategy to stabilize VSMCs and favorably influence atherosclerotic plaque biology.

**Abstract:**

Genome-wide association studies have uncovered many coronary artery disease (CAD) loci, but mechanisms linking risk variants to vascular biology remain unclear. We screen candidate causal CAD genes for regulators of the vascular smooth muscle cell (VSMC) contractile phenotype using a pooled CRISPR–Cas9 knockout library in primary human VSMCs with MYH11 protein levels as the readout. FHL3 (four-and-a-half LIM domains protein 3) emerges as the top repressor of the contractile state. Colocalization analyses in a VSMC biobank and vascular tissues associate a CAD risk allele with reduced *FHL3* expression. Biochemical and imaging studies show FHL3 is associated and co-localizes with the transcriptional co-activator MRTFB (myocardin-related transcription factor-B). Functionally, *FHL3* overexpression attenuates MRTFB-serum response factor (SRF)-driven induction of contractile markers (MYH11, transgelin), whereas *FHL3* knockdown increases their expression in an MRTFB-dependent manner. *FHL3* knockdown or *MRTFB* overexpression reduces VSMC proliferation and migration, and *FHL3* overexpression mitigates MRTFB-driven effects on these behaviors. These data identify FHL3 as a regulator of VSMC plasticity that inhibits the contractile phenotype by interfering with MRTFB-SRF signaling, link a CAD-associated genetic signal to the VSMC phenotype, and nominate the FHL3-MRTFB interaction as a potential therapeutic target.

## 1. Introduction

Atherosclerotic diseases, particularly coronary artery disease (CAD), are a leading cause of death in many countries [1]. In recent years, genome-wide association studies (GWAS) have found a panel of genetic loci associated with CAD risk [2]. A key step for potential therapeutic translation of these genetic discoveries is to identify the causal genes at these loci and understand their pathobiological roles. Notably, a recently reported integrative analysis of arterial tissue single-cell transcriptomic and CAD GWAS data reveals that the genes residing at the CAD risk loci are enriched in vascular smooth muscle cells (VSMCs) [3]. Furthermore, our recent studies implementing functional genomics analyses of a large VSMC bank in conjunction with CAD GWAS data have identified candidate causal genes at many of the CAD loci [4,5].

VSMCs play a multifaceted role in atherosclerosis [6,7]. In contrast to skeletal muscles and cardiomyocytes, which are terminally differentiated cells, VSMCs possess phenotypic plasticity [8,9]. In atherogenesis, VSMCs de-differentiate from a contractile state to a proliferative phenotype and migrate into the atherosclerotic lesion [8,9]. While they contribute to the formation and growth of the atherosclerotic plaque, their accumulation in the fibrous cap helps stabilize the atherosclerotic plaque, reducing the risk of plaque rupture and consequent coronary ischemic events [6,7].

Contractile VSMCs are typically characterized by the expression of certain markers, such as smooth muscle myosin heavy chain 11 (MYH11) and transgelin (TAGLN) [8,9]. The transcription of these genes is activated by the transcription factor serum response factor (SRF) together with its co-activators myocardin, myocardin-related transcription factor-A (MRTFA) and myocardin-related transcription factor-B (MRTFB) [8,10].

In the present study, we investigated whether and which candidate causal CAD genes we recently identified [4,5] could modulate the VSMC phenotype. To this end, we performed a pooled CRISPR-Cas9 nuclease knockout screen targeting the candidate causal CAD genes in human VSMCs, using MYH11 (smooth muscle myosin heavy chain 11) levels as a readout for the phenotypic state. FHL3 (four-and-a-half LIM domains protein 3) was identified as the top-ranked repressor of the contractile phenotype marked by high MYH11 levels. Further experiments showed that FHL3 complexed with MRTFB and inhibited the up-regulatory effect of MRTFB and SRF on MYH11 and TAGLN expression, therefore identifying a FHL3-MRTFB/SRF axis that modulates the VSMC phenotype.

## 2. Materials and Methods

The study was conducted in accordance with the Declaration of Helsinki (1975, revised in 2013). The VSMC bank study described below was approved by the East Midlands—Derby Research Ethics Committee (15/EM/0045, 20/EM/0028 and 25/EM/0038) on 20 May 2015, 22 April 2020 (renewal) and 6 May 2025 (renewal), and the parents of umbilical cord donors gave written informed consent.

### 2.1. VSMC Biobank, Correlation Analyses, Genetic Colocalization Analysis, and Gene Expression Levels by Genotype Analysis

A collection of VSMCs from different individuals [4] was utilized in this study. As described before [4], VSMCs were isolated from umbilical cord arteries of 2114 donors using a previously established protocol [11]. DNA from 1992 samples was genotyped using the Illumina Infinium Global Screening Array-24 v2.0, covering approximately 760,000 variants, followed by genotype imputation to obtain information for 7,334,165 variants. RNA sequencing was performed on total RNA extracted from passage 3 VSMCs from 1499 donors. Strand-specific, rRNA-depleted libraries were prepared and sequenced using 150-bp paired-end reads on an Illumina platform at a depth of approximately 30 million reads per sample. Data of both genotype and RNA sequencing were available from 1486 subjects (780 males and 706 females) for subsequent analyses.

eQTL analysis was conducted in 1486 samples with both genotype and transcriptomic data, using linear additive regression to model the effect of each genetic variant located within 1 Mb of the transcription start site of each gene with the R package MatrixEQTL [12]. Sex, the first 2 principal components and the first 10 PEER (probabilistic estimation of expression residuals) factors were used as covariates. Significance was determined using the eigenMT-BH multiple testing correction method [13].

To test for colocalization between CAD GWAS [14,15,16,17] and VSMC *cis*-eQTL, we previously performed SMR/HEIDI analysis [4,5]. We ran the SMR/HEIDI analysis with the default setting, which tests a genomic window size of 2 Mb centered around the gene of interest and considered a single causal variant [18]. We applied a threshold of p_SMR_ < 0.05 & p_HEIDI_ > 0.05 to identify significant colocalizations of CAD GWAS signals with VSMC eQTL signals.

Functional characterization of passage 3 VSMCs included proliferation, migration, and apoptosis assays performed using the Operetta CLS High-Content Analysis System (PerkinElmer, Waltham, MA, USA).

With the use of the above data, correlation analyses were performed with the *cor* function in R (v4.3.1) to determine the correlation of *FHL3* expression levels with *MYH11* expression levels and with VSMC proliferation, migration, and apoptosis. Correlation plots were generated with R package ggplot2 (v4.0.3). Genetic colocalization plots were generated with the LocusCompare R package (v1.1.0) [19]. *FHL3* expression levels by genotype were plotted in R.

### 2.2. Pooled CRISPR-Cas9 Nuclease Knockout Screen

A pooled CRISPR-Cas9 knockout screen was performed targeting 101 protein-coding genes identified by colocalization analyses as putative causal genes for coronary artery disease (CAD) in our previous studies [4,5]. For each target gene, four single-guide RNAs (sgRNAs) were selected from the Human CRISPR Knockout Pooled Library (GeCKO v2 or Brunello), together with non-targeting control sgRNAs. Corresponding oligonucleotides were synthesized (GenScript, Nanjing, China) and cloned into the lentiGuide-Puro vector (Addgene, Watertown, MA, USA; #52963), as previously described [20]. The sgRNA library was amplified and packaged into lentivirus according to established protocols [20]. Library quality and representation were evaluated by next-generation sequencing and analyzed using MAGeCK (Model-based Analysis of Genome-wide CRISPR-Cas9 Knockout) [21].

Human aortic smooth muscle cells (HASMCs; Lonza, CC-2571) were seeded in 15-cm culture dishes at a density of 2 × 10^6^ cells per dish. A total of 1 × 10^7^ cells per technical replicate were transduced with the pooled sgRNA lentiviral library at a multiplicity of infection (MOI) of 0.2 (the experiment included 2 technical replicates). Forty-eight hours after infection, cells were selected with 1 μg/mL puromycin for 7 days to enrich successfully transduced cells. Following antibiotic selection, cells were cultured in puromycin-free medium for an additional 48 h before harvesting.

For Cas9-mediated genome editing, cells were pelleted and resuspended in Neon™ NxT Electroporation Buffer R (ThermoFisher Scientific, Waltham, MA, USA; N10096) at a concentration of 1 × 10^6^ cells per 100 μL. Recombinant Cas9 protein (ThermoFisher Scientific, A36499) was added at a 1:10 (*v*/*v*) ratio, and electroporation was performed using the Neon™ NxT Electroporation System (Thermo Fisher Scientific, Waltham, MA, USA; MPK5000) at 1 × 10^6^ cells per reaction (pulse voltage, 1475 V; pulse width, 20 ms; pulse number, 2). The total number of electroporations was scaled according to experimental requirements. Immediately after electroporation, cells were transferred to pre-warmed culture medium and allowed to recover. The efficiency of Cas9-mediated editing is shown in Supplemental Figure S1A.

Nine days after electroporation, cells were harvested, washed with phosphate-buffered saline (PBS), and fixed/permeabilized using fixation/permeabilization buffer (ThermoFisher Scientific, 00-5523-00) for 30 min. Cells were incubated with rabbit anti-MYH11 antibody (Abcam, Cambridge, UK; ab224804) for 1 h, followed by Alexa Fluor 488-conjugated goat anti-rabbit IgG H&L secondary antibody (Abcam, ab150077) for 30 min. Between staining steps, cells were washed twice with PBS supplemented with 1% fetal bovine serum. Stained cells were filtered through a 40 μm cell strainer and resuspended at a final concentration of 2 × 10^6^ cells/mL. Fluorescence-activated cell sorting gates were established based on Alexa Fluor 488 fluorescence intensity to isolate the top 15% and bottom 15% of MYH11-expressing cells. Genomic DNA was subsequently extracted from sorted cell pellets as previously described [22].

sgRNA barcode sequences were amplified from genomic DNA using Q5 Hot Start High-Fidelity 2× Master Mix (New England Biolabs, Ipswich, MA, USA; M0494L). Polymerase chain reaction (PCR) primers were designed according to the protocol reported by Joung et al. [23]. PCR amplification was performed under the following conditions: 95 °C for 5 min; 24–26 cycles of 98 °C for 20 s, 60 °C for 15 s, and 72 °C for 15 s, followed by a final extension at 72 °C for 1 min. PCR products were pooled and purified using Hieff NGS^®^ DNA Selection Beads (Yeasen Biotechnology, Shanghai, China; 12601ES08). Purified libraries were eluted in 50 μL nuclease-free water, quantified using the Qubit dsDNA HS Assay Kit (ThermoFisher Scientific, Q32851), and assessed for quality using a NanoDrop spectrophotometer (Thermo Fisher Scientific, Waltham, MA, USA).

Next-generation sequencing at 400 × coverage per sgRNA was performed to determine the abundance of individual sgRNAs. Raw sequencing reads were processed using Cutadapt (V4.8) [24] for adapter trimming, together with custom bioinformatics scripts, achieving a mapping rate > 98%. The distribution of sgRNA counts and reproducibility between replicates are shown in Supplemental Figure S2B,C. Enrichment and depletion analyses were conducted using the MAGeCK software package (version 0.5.9.5) [21]. MAGeCK count and test algorithms were applied with normalization to non-targeting control sgRNAs to identify positively and negatively selected sgRNAs associated with MYH11 expression. The results for the 4 individual *FHL3*-targeting sgRNAs in each technical replicate are shown in Figure S1D.

### 2.3. Bioinformatics Analyses

#### 2.3.1. Atherosclerotic Plaque Single-Cell RNA-Sequencing Data Analysis

Single-cell RNA-sequencing data from human thoracic aorta were obtained from the NCBI Gene Expression Omnibus (GEO) under accession GSE253934 (Homo sapiens; expression profiling by high-throughput sequencing). The processed count matrices and associated metadata were downloaded from the GEO supplementary files (GSE253934_RAW.tar).

Filtered gene–barcode matrices generated by 10 × Genomics Cell Ranger (v9.0.1)were uploaded to Trailmaker (Parse Biosciences, Seattle, WA, USA; https://app.trailmaker.parsebiosciences.com) for primary downstream analysis. Filtered feature–barcode matrices were generated and subsequently used for quality control, processing, exploration, and visualization. Quality control filters were applied to remove barcodes corresponding to: (i) potential dying, dead, or stressed cells with high mitochondrial transcript content, using a per-sample threshold of 15%; (ii) outliers in the relationship between the number of detected genes and total transcript counts, identified by linear regression (*p* < 4.19 × 10^−4^–1.00 × 10^−3^, per sample); and (iii) cells with a high probability of being doublets (doublet score threshold > 0.526–0.787, per sample).

After quality filtering, datasets were integrated in Seurat (V4) using Harmony with 2000 highly variable genes (LogNormalize), followed by dimensionality reduction using RPCA (30 PCs) and graph-based clustering using the Leiden algorithm (resolution = 0.8) to generate the initial cell atlas for downstream analyses. UMAP embeddings were computed using cosine distance with min.dist = 0.4. For comparison of genes of interest, normalized expression values for the relevant clusters were exported using the normalized gene expression function in Trailmaker.

#### 2.3.2. Arterial Tissue eQTL Data and Genetic Colocalization Analysis

eQTL data from coronary artery, aorta, and tibial artery tissues, respectively, were downloaded from the GTEx portal https://gtexportal.org/home/ (accessed on 15 August 2024) [25]; CAD GWAS summary statistics from Aragam et al. [14] were obtained from http://ftp.ebi.ac.uk/pub/databases/gwas/summary_statistics/GCST90132001-GCST90133000/GCST90132314 (accessed on 26 February 2026). Coloc [26] was performed to examine colocalization of CAD GWAS signals with *FHL3* eQTL signals in coronary artery, aorta, and tibial artery tissues. We applied a threshold of PP.H4.abf value of >0.8 for statistical significance for a positive colocalization via the same variant. Genetic colocalization plots were generated using LocusCompare R package (v4.3.2) [19].

#### 2.3.3. Protein–Protein Interaction Analyses

Protein–protein interaction networks were constructed using the STRING database (version 11.0, https://string-db.org (accessed on 5 November 2025)) [27]. Interactions with a combined confidence score > 0.4 (medium confidence) were retained in full STRING network. The resulting network was exported for visualization and further analysis.

Additionally, BioGRID (Biological General Repository for Interaction Datasets, https://thebiogrid.org/ (accessed on 28 October 2025)) [28] was interrogated for data indicating a protein–protein interaction between FHL3 and MRTFB.

### 2.4. Cell Culture

HASMCs were maintained in Human Vascular Smooth Muscle Cell Basal Medium (ThermoFisher Scientific, M231500) supplemented with Smooth Muscle Growth Supplement (ThermoFisher Scientific, S00725) and 1 × penicillin–streptomycin. Cells were cultured at 37 °C under humidified conditions in an atmosphere containing 5% CO_2_.

### 2.5. Lentivirus Generation and Transduction

We constructed gene overexpression and knockdown vectors using the lentiviral vectors pLVX-puro (Clontech Laboratories, Inc., Mountain View, CA, USA; 632164) and pLKO.1-puro (Addgene, #8453), respectively. For overexpression, the cDNA sequences were obtained from GenBank/NCBI with accession numbers 30678.1 for *FHL3* and 76823.1 for *MRTFB*, respectively. For gene knockdown, the short hairpin RNA (shRNA) sequences were CCCTTAAATCTAGACTTCTCT (sh-FHL3) for *FHL3*; CCAGTGTTAAAGAAGCAATTA (sh-MRTFB) for *MRTFB*; and TAAGTCGCCCTCGCTCGAG (sh-NC) for a negative control vector.

HEK293T cells (ATCC, CRL-3216) were co-transfected with the lentiviral expression vectors described above, together with the packaging plasmid psPAX2 (Addgene, #12260) and the envelope plasmid pMD2.G (Addgene, #12259), using Lipofectamine 3000 (Invitrogen, Carlsbad, CA, USA; L3000015) according to the manufacturer’s instructions. Following transfection, cells were maintained in Dulbecco’s Modified Eagle Medium (DMEM) supplemented with 10% fetal bovine serum. Lentiviral supernatants were harvested at 24 and 48 h post-transfection, pooled, and stored at −80 °C until use.

For lentiviral transduction, HASMCs at approximately 70% confluence were incubated with 750 μL of lentiviral supernatant in the presence of 10 ng/mL polybrene. The viral medium was replaced with fresh culture medium after 24 h. Forty-eight hours after transduction, puromycin was added to the culture medium at a final concentration of 1.0 μg/mL for antibiotic selection. Puromycin-resistant cells were subsequently expanded for downstream experiments.

### 2.6. Western Blot Analyses

HASMC lysates were prepared using radioimmunoprecipitation assay (RIPA) buffer, and protein concentrations were quantified using the Pierce BCA Protein Assay Kit (ThermoFisher Scientific, 23227). Equal amounts of protein were resolved by sodium dodecyl sulfate–polyacrylamide gel electrophoresis and transferred onto polyvinylidene difluoride membranes. Membranes were incubated with the appropriate primary antibodies followed by horseradish peroxidase-conjugated secondary antibodies (Supplemental Table S1). Immunoreactive protein bands were detected and visualized using the ChemiDoc XRS+ Imaging System (Bio-Rad Laboratories, Hercules, CA, USA). Vinculin (~117 kDa) or α-tubulin (~55 kDa) was used as a loading control, selected based on molecular weight compatibility with the target proteins to avoid signal overlap and ensure accurate normalization.

### 2.7. Co-Immunoprecipitation

HASMC lysates were prepared as described above and incubated with an anti-FHL3 antibody, an anti-MRTFB antibody, or a control IgG (Supplemental Table S1), followed by incubation with Pierce Protein A/G Magnetic Beads (ThermoFisher Scientific, #88802). After the washing steps, the supernatants containing the target antigens were analyzed by Western blot, as described above.

### 2.8. Dual Luciferase Reporter Gene Assays

The promoter regions of the human *MYH11* and *TAGLN* genes were amplified by PCR and subcloned into the pGL3-basic vector (Promega, E1751), as previously described [29]. Each of these constructs, together with the pRL-TK control plasmid (Promega, E2241), were transfected into HASMCs, and luciferase activity was measured 2 days post-transfection using the FilterMax F5 (Molecular Devices, San Jose, CA, USA). The firefly luciferase activity reading was normalized to the *Renilla* luciferase activity for each sample.

### 2.9. Immunofluorescence Analysis

HASMCs were fixed with 4% paraformaldehyde, permeabilized with 0.3% Triton X-100, and blocked with 3% bovine serum albumin. Cells were then incubated overnight at 4 °C with primary antibodies, followed by incubation with Alexa Fluor 488- or Alexa Fluor 594-conjugated secondary antibodies for 1 h at room temperature (Supplemental Table S1). After mounting with antifade medium containing 4′,6-diamidino-2-phenylindole (DAPI), fluorescent images were acquired using a Zeiss confocal microscope (Carl Zeiss AG, Oberkochen, Germany).

### 2.10. Proliferation Assay

HASMCs were seeded into 96-well plates at a density of 2000 cells per well. After 48 h of culture, cell proliferation was assessed using the Cell Counting Kit-8 (Beyotime, C0038) according to the manufacturer’s instructions. Absorbance was measured at 450 nm using a microplate reader (Molecular Devices, San Jose, CA, USA).

### 2.11. Migration Assay

HASMCs (4 × 10^4^ cells/well) were seeded into the Transwell well (with 8 μm pores) of a Corning Transwell 24-well plate (Corning, NY, USA; CLS3422-48EA). After 24 h, the cells were fixed with 4% formaldehyde (Sigma-Aldrich, F8775-500ML) and then stained with 1 μg/mL Hoechst 33342 (Yeasen, 40732ES03). Next, non-migrated cells on the upper surface of the Transwell membrane were carefully removed, and five images of the lower surface were acquired using a fluorescence microscope with a 10× objective lens. The number of migrated cells in each image was quantified using ImageJ software (V1.52a), and the mean value from the five images was calculated.

### 2.12. Apoptosis Assay

HASMCs were stained with annexin V-fluorescein isothiocyanate (Annexin V-FITC) and propidium iodide, respectively, using a Dead Cell Apoptosis Kit (Yeasen, 40302ES50) according to the manufacturer’s instructions. The cells were subsequently analyzed using a Sparrow Flow Cytometer (Celula Inc., San Diego, CA, USA).

### 2.13. Statistical Analyses

The eQTL analysis, genetic colocalization analyses and pooled CRISPR knockout screening data analysis are described above. A total of linear correlation analyses was performed to test relationships of the *FHL3* expression level with the *MYH11* expression level or with data of VSMC proliferation (EdU intensity), migration speed, migration distance, and apoptosis, respectively. ANOVA (analysis of variance) was performed to compare *FHL3* expression levels between genotypes for rs28428561. In the experiments of HASMCs with targeted gene knockdown or overexpression, non-parametric tests (Mann–Whitney U test for two-group comparisons and Kruskal–Wallis test followed by Dunn’s post hoc test for multiple group comparisons) were performed, because the sample sizes were relatively small and normality could not be assumed. A two-sided *p* value < 0.05 was considered statistically significant.

## 3. Results

### 3.1. Identification of FHL3 as a Repressor of VSMC Contractile Marker Genes

**Figure 1:**
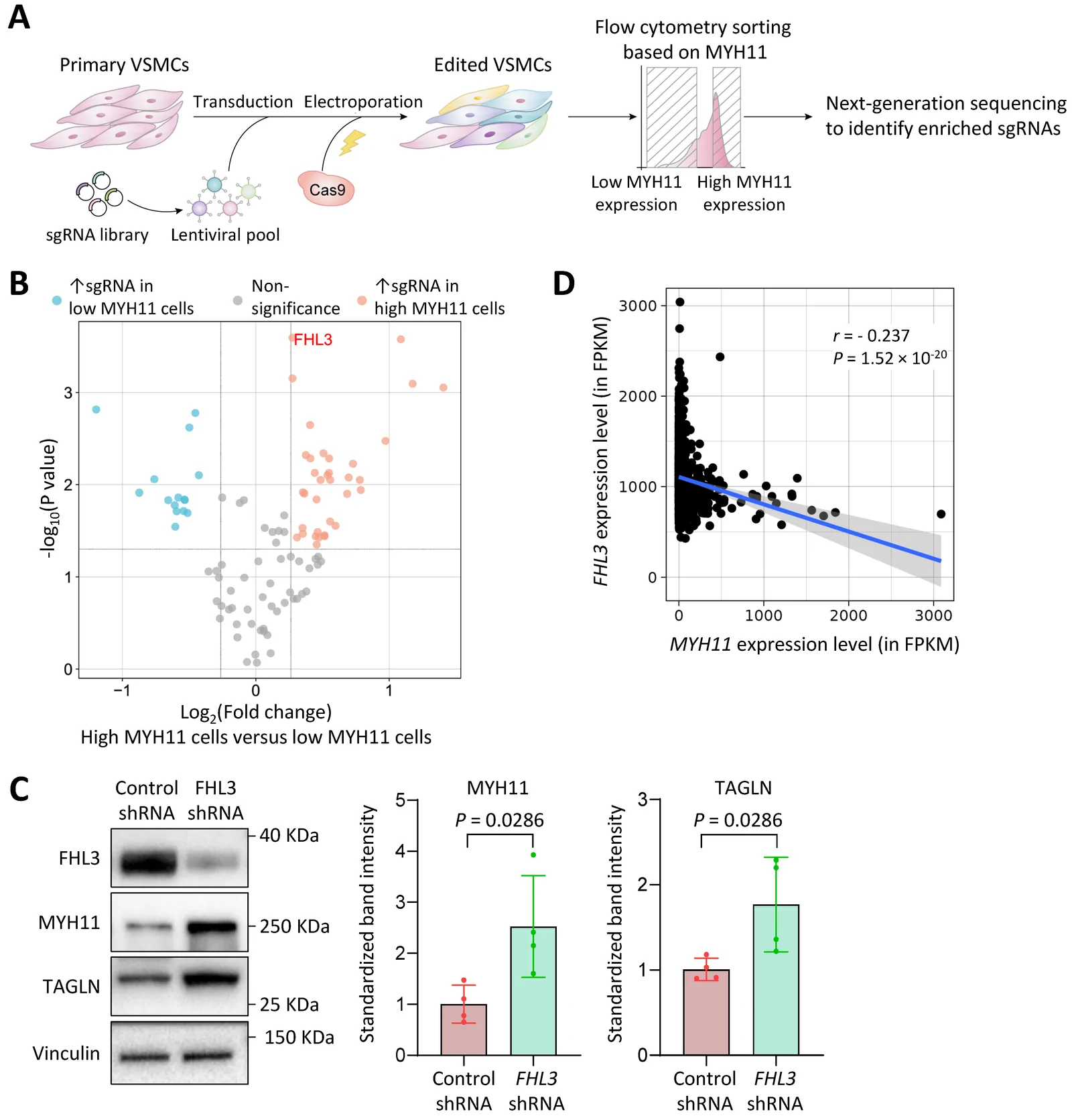
*FHL3* represses contractile VSMC marker gene expression. (**A**) A schematic illustration of the pooled CRISPR-Cas9 nuclease knockout screen experimental procedure. Abbreviations: clustered regularly interspaced short palindromic repeats, CRISPR; CRISPR-associated protein 9, Cas9; single-guide RNA, sgRNA. (**B**) Volcano plot showing results of the pooled CRISPR-Cas9 nuclease knockout screen. Pink symbols indicate genes whose knockout increases MYH11 in VSMCs. (**C**) VSMCs were transduced with either a lentiviral vector containing a *FHL3*-targeting shRNA or a non-targeting control shRNA, followed by Western blot analysis of FHL3, MYH11, and TAGLN. Representative Western blot images are presented on the left. Column charts show means (±standard deviation) of MYH11 and TAGLN band intensities standardized against the vinculin band. Each plotted data point represents a technical replicate. *p* < 0.05 from two-sided Mann–Whitney U test, *n* = 4 technical replicates per condition. (**D**) Scatter plot showing an inverse correlation between *FHL3* and *MYH11* expression levels in VSMCs from different individuals from a VSMC biobank that we recently developed [4]. Each plotted data point represents an independent biological replicate (donor-derived VSMC sample). *p* value from linear correlation analysis, *n* = 1499 biological replicates for both *FHL3* and *MYH11* expression data. FPKM: fragments per kilobase of transcript per million mapped reads; *r*: correlation coefficient.

A pooled CRISPR-Cas9 nuclease knockout screen was performed on 101 candidate causal genes for CAD identified by genetic colocalization analyses in our recent study [4]. In this experiment, cultured VSMCs were transduced with a sgRNA library including sgRNAs to target these genes and then subjected to fluorescence-activated cell sorting with the use of an antibody for MYH11. Following the flow cytometry sorting, either the top or bottom 15% cells based on MYH11 expression levels were subjected to next-generation sequencing (outlined in Figure 1A).

The *FHL3* gene ranked highest among the genes for which sgRNA-mediated knockout was detected in the high-MYH11 cell subpopulation (Figure 1B and Supplemental Table S2). Following the CRISPR screen, we focused our study on *FHL3*. A validation experiment showed that short hairpin RNA (shRNA)-mediated knockdown of *FHL3* resulted in increased levels of MYH11 and also TAGLN (Figure 1C), supporting the finding of the CRISPR screen.

In further support, we observed an inverse correlation between *FHL3* and *MYH11* expression levels in VSMCs from different individuals (*n* = 1486) (Figure 1D).

### 3.2. FHL3 Expression in VSMCs Is Influenced by CAD-Associated Genetic Variants

**Figure 2:**
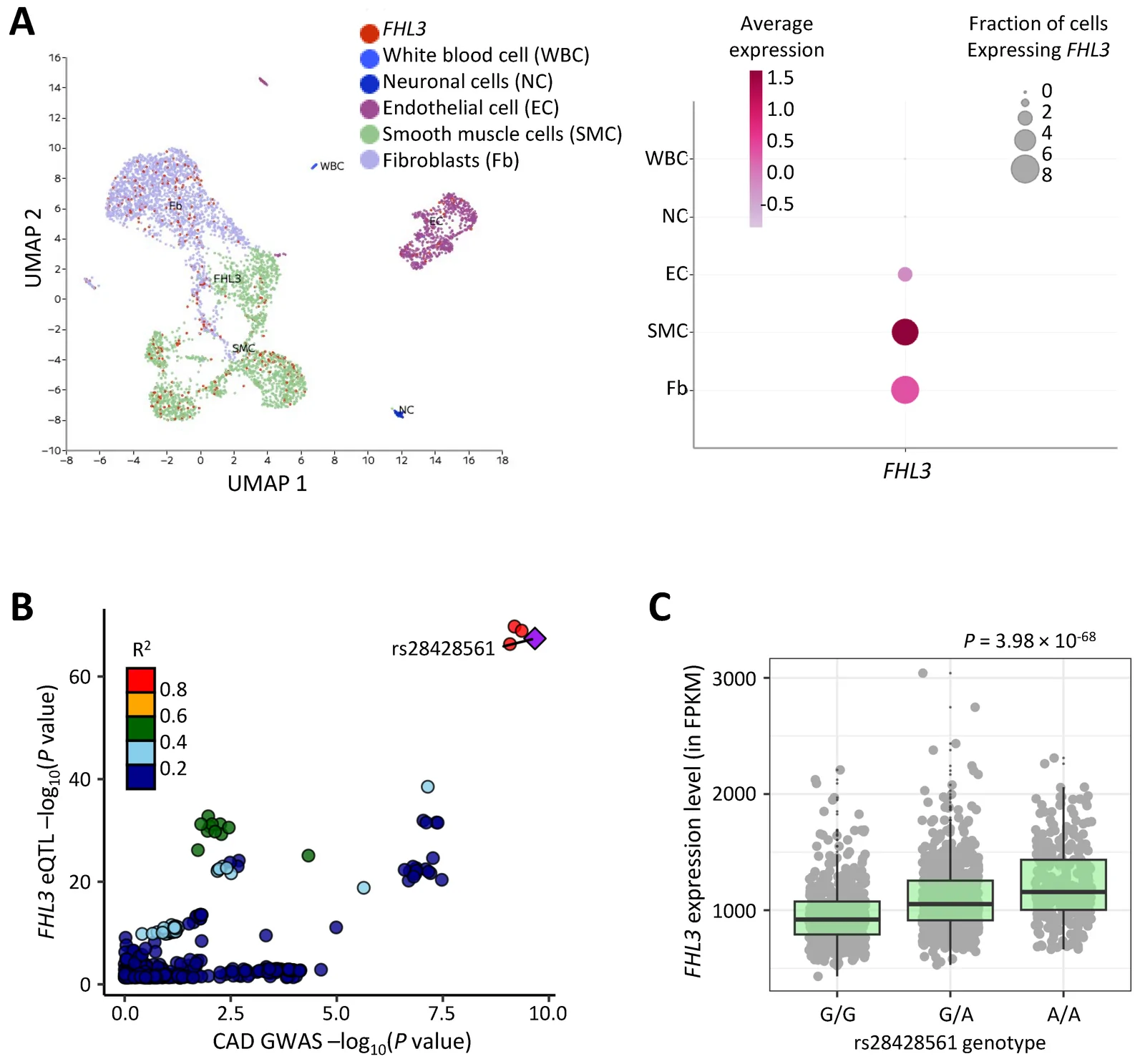
Influence of CAD-associated genetic variant on *FHL3* expression in VSMCs. (**A**) UMAP (Uniform Manifold Approximation and Projection) visualization (left) and bubble plot (right) of *FHL3* gene expression in various cell types in atherosclerotic arterial tissues (data from GSE253934 [31]), showing enrichment of FHL3 in vascular smooth muscle cells (SMC). (**B**) Scatter plot showing colocalization of CAD GWAS with VSMC *FHL3* eQTL signals, supporting *FHL3* as a candidate effector gene at this CAD locus. *n* = 1486 biological replicates for *FHL3* expression data and *n* = 1,165,690 biological replicates for CAD GWAS data. (**C**) Box and whisker plot of *FHL3* expression levels in VSMCs by genotype of the CAD-associated genetic variant rs28428561. Each plotted data point represents an independent biological replicate (donor-derived VSMC sample). Each box shows the median, first quartile and third quartile, while each whisker line shows the minimum and maximum values excluding outliers. *p* value from ANOVA (analysis of variance); *n* = 1486 biological replicates for both rs28428561 genotype and *FHL3* expression data.

An analysis of single-cell transcriptomic data from a previously reported studies [30,31] showed that in atherosclerotic or aneurysmal arterial tissues, VSMCs express higher levels of *FHL3* than fibroblasts and endothelial cells (Figure 2A and Supplemental Figure S2). In contrast, in healthy vascular tissues, VSMCs express lower levels of *FHL3* than fibroblasts and endothelial cells (Supplemental Figure S3). Genes reported to be co-expressed with *FHL3* are shown in Supplemental Figure S4.

In a genetic colocalization analysis of gene expression data from the abovementioned VSMC biobank that we developed [4], in conjunction with reported CAD GWAS data [14], we previously detected genetic variants with an eQTL effect on *FHL3* expression in VSMCs, as well as a significant association with CAD (*p* = 3.4 × 10^−9^ for rs4072980 in SMR analysis) [5], supporting *FHL3* as a candidate effector gene at this CAD locus. The colocalization can be visualized in the LocusCompare plot in Figure 2B. The highlighted variant rs28428561 in this plot is in high linkage disequilibrium with rs4072980 (R^2^ = 0.98 in Europeans and R^2^ = 0.96 in East Asians). In this VSMC biobank, *FHL3* expression was significantly lower in cells of the rs28428561 G/G genotype than in cells of the A/A genotype and intermediate in cells of the G/A genotype (Figure 2C). Of note, the rs28428561 G-allele is in linkage disequilibrium (R^2^ = 0.97) with the G-allele of rs4072980 that is reported to be associated with higher CAD risk in multiple GWASs [14,15,16].

**Figure 3:**
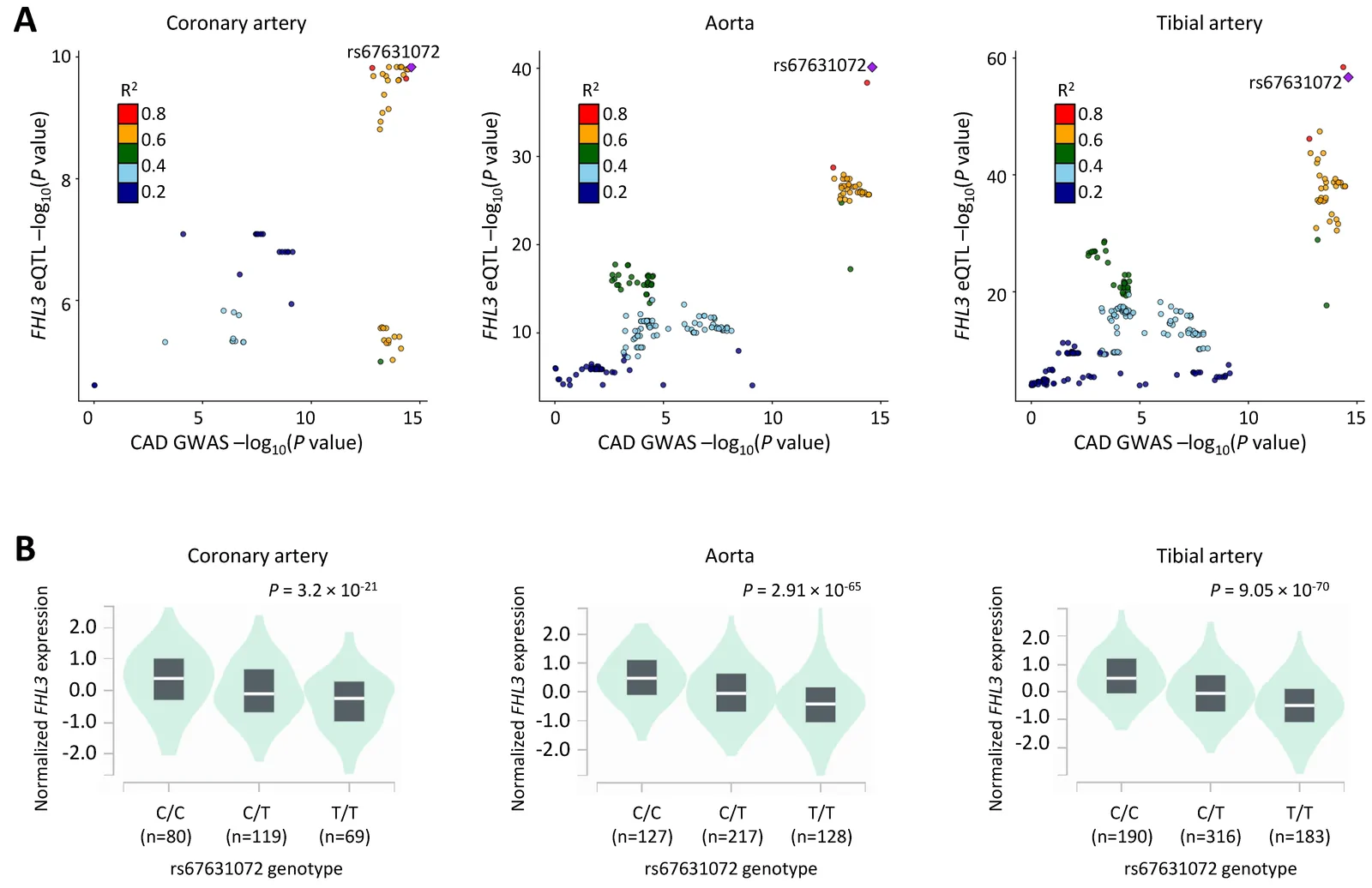
Influence of CAD-associated genetic variant on *FHL3* expression in arterial tissues. (**A**) Scatter plot showing colocalization of CAD GWAS with arterial tissue *FHL3* eQTL signals, supporting *FHL3* as a candidate effector gene at this CAD locus. *n* = 268 (for coronary artery), 472 (for aorta) and 689 (tibial artery) biological replicates for *FHL3* expression data and *n* = 1,165,690 biological replicates for CAD GWAS data. (**B**) Scatter plot of *FHL3* expression levels in arterial tissues by genotype of the CAD-associated genetic variant rs67631072. The numbers of biological replicates are indicated in the figures.

Furthermore, an analysis of data from the Genotype-Tissue Expression project (GTEx) also showed colocalization of *FHL3* eQTL and CAD GWAS signals in arterial tissues, including the coronary artery, aorta, and tibial artery (PP.H4.abf value of >0.8 for each tissue-type) (Figure 3A and Supplemental Tables S1–S3). The CAD-associated rs67631072 T-allele [14,15,16] (in linkage disequilibrium with the rs28428561 G-allele, R^2^ = 0.86) was associated with lower *FHL3* expression in these arterial tissues (Figure 3B).

### 3.3. FHL3 Interacts with MRTFB

**Figure 4:**
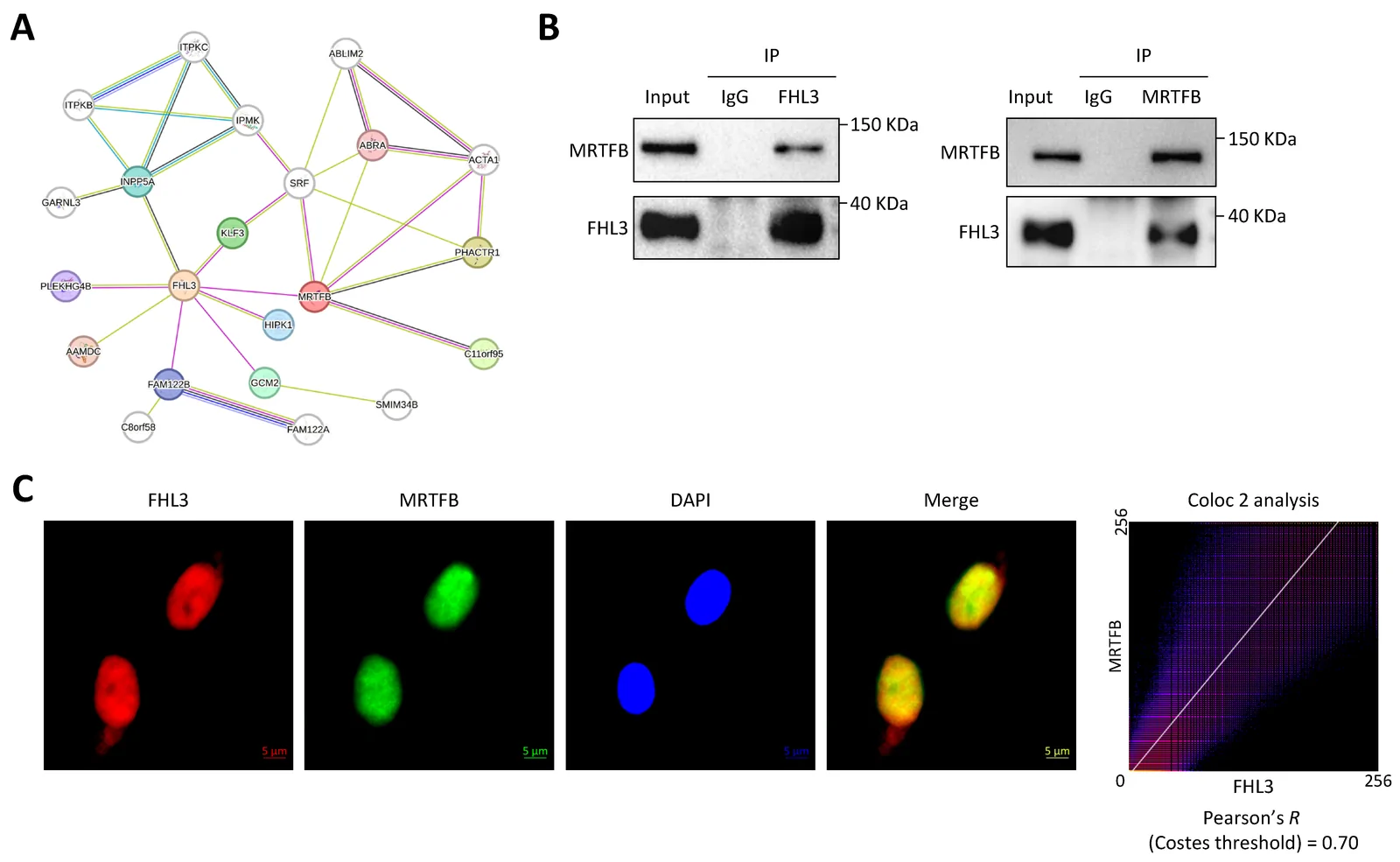
FHL3 is associated with MRTFB in VSMCs. (**A**) A schematic illustration of a reported association between FHL3 and MRTFB based on STRING network analysis. (**B**). Results of co-immunoprecipitation assay showing that FHL3 and MRTFB are present within the same protein complex in VSMCs. Cell lysates were subjected to immunoprecipitation using either an anti-FHL3 antibody (left) or an anti-MRTFB antibody (right), followed by Western blot analysis using either the anti-FHL3 antibody or the anti-MRTFB antibody, as indicated in the figure. (**C**). Fluorescent immunocytochemical analysis showing subcellular colocalization of FHL3 (red) and MRTFB (green) in VSMCs. Nuclei are visualized by DAPI staining. Images shown are representative of three independent experiments. Colocalization was quantified using Pearson’s correlation coefficient with Costes automatic thresholding using the Coloc 2 plugin in Fiji.

An interrogation of the STRING (Search Tool for the Retrieval of Interacting Genes/Proteins) and BioGRID (Biological General Repository for Interaction Datasets) databases indicated that FHL3 could interact with MRTFB (Figure 4A), an SRF coactivator that has been reported to promote the contractile phenotype of VSMCs [10,32]. To determine whether FHL3 and MRTFB are present in the same protein complex in VSMCs, we performed a co-immunoprecipitation assay. In this assay, we detected MRTFB in immunoprecipitates pulled down using an anti-FHL3 antibody and conversely detected FHL3 in immunoprecipitates pulled down using an anti-MRTFB antibody (Figure 4B), supporting an association between FHL3 and MRTFB in VSMCs. In further support, confocal microscopic examination of VSMCs immunostained for both FHL3 and MRTFB showed co-localization of these two proteins in VSMC nuclei (Figure 4C and Supplemental Figure S5).

### 3.4. FHL3 Represses Contractile VSMC Marker Gene Expression via the MRTFB-SRF Pathway

**Figure 5:**
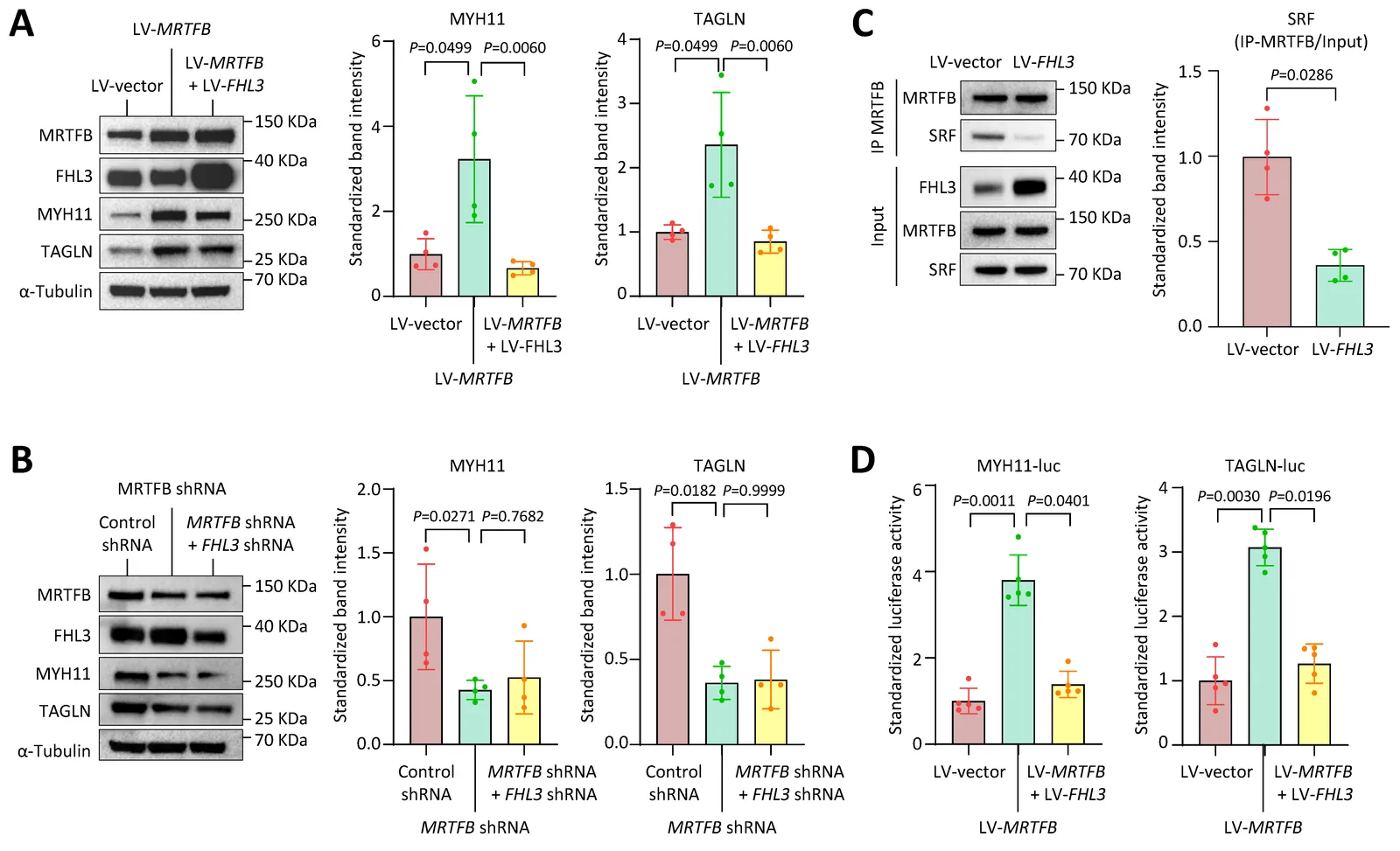
FHL3 inhibits contractile VSMC marker gene expression by interfering MRTFB-SRF interaction. (**A**) VSMCs were transduced with either an empty lentiviral vector (LV-vector) to serve as a control, a lentiviral vector to express *MRTFB* (LV-*MRTFB*), or lentiviral vectors to express *MRTFB* (LV-*MRTFB*) and *FHL3* (LV-*FHL3*), respectively, followed by Western blot analysis of MYH11 and TAGLN. (**B**) VSMCs were transduced with either a lentiviral vector to express a non-targeting short hairpin RNA (shRNA) to serve as a control, a lentiviral vector to express a shRNA to target *MRTFB* (*MRTFB* shRNA), or lentiviral vectors to express shRNAs to target *MRTFB* (*MRTFB* shRNA) and *FHL3* (*FHL3* shRNA), respectively, followed by Western blot analysis of MYH11 and TAGLN. (**C**) VSMCs transduced to either an empty lentiviral vector (control) or a lentiviral vector to express *FHL3*, were subjected to immunoprecipitation using an anti-MRTFB antibody, followed by Western blot analysis with antibodies against either MRTFB or SRF, as indicated in the figure. SRF band intensity was normalized to the corresponding SRF input signal. (**A**–**C**) Representative Western blot images are presented on the left. Column charts show mean (±standard deviation) standardized band intensities. Each plotted data point represents a technical replicate. *p* values from two-sided Mann–Whitney U test or two-sided Kruskal–Wallis test with post hoc Dunn’s test, *n* = 4 technical replicates per condition. (**D**) VSMCs were transduced with either an empty lentiviral vector (control), a lentiviral vector to express *MRTFB*, or lentiviral vectors to express *MRTFB* and *FHL3*, and transfected with luciferase reporter plasmids containing either the *MYH11* gene promoter (MYH11-luc) or the *TAGLN* gene promoter (TAGLN-luc), followed by luciferase activity assays. Column charts show means (±standard deviation) of luciferase activity. Each plotted data point represents a technical replicate. *p* values from two-sided Kruskal–Wallis test with post hoc Dunn’s test; *n* = 4 technical replicates per condition.

Next, we investigated whether FHL3 modulates the VSMC contractile phenotype via MRTFB. We found that *MRTFB* overexpression in VSMCs resulted in a significant increase in both MYH11 and TAGLN levels. Importantly, this up-regulatory effect was abolished by concurrent overexpression of *FHL3* (Figure 5A), suggesting that FHL3 inhibits the up-regulating effect of MRTFB on the expression of these contractile VSMC hallmark proteins.

In support, shRNA-mediated knockdown of *MRTFB* resulted in a substantial reduction of both MYH11 and TAGLN, and concurrent knockdown of *FHL3* did not lead to an otherwise expected up-regulation of these proteins (Figure 5B).

Since MRTFB functions as an SRF co-activator [10,33], we investigated whether FHL3 influences the association between MRTFB and SRF A co-immunoprecipitation assay detected SRF in immunoprecipitates pulled down by an anti-MRTFB antibody (Figure 5C), confirming the interaction between MRTFB and SRF. Importantly, the assay showed that in VSMCs with FHL3 overexpression, SRF diminished in immunoprecipitates pulled down by the anti-MRTFB antibody (Figure 5C), suggesting that FHL3 overexpression reduced the association between MRTFB and SRF detected by co-immunoprecipitation.

As it has been reported that MRTFB enhances SRF binding to CArG box elements in the promoter regions of VSMC contractile genes and thereby up-regulates their expression [33], we investigated whether FHL3 could counteract the up-regulatory effect of MRTFB on VSMC contractile gene promoter activity. To this end, we transfected VSMCs with luciferase reporter gene plasmid vectors containing the promoter regions of *MYH11* and *TAGLN*, respectively, as well as either an empty lentiviral vector, a *MRTFB*-expressing lentiviral vector, or a *MRTFB*-expressing lentiviral vector plus a *FHL3*-expressing lentiviral vector, followed by a luciferase activity assay. The assay showed that *MRTFB* overexpression increased *MYH11* and *TAGLN* gene promoter activity as expected and, importantly, *FHL3* overexpression significantly attenuated the up-regulatory effect of MRTFB on *MYH11* and *TAGLN* gene promoter activity (Figure 5D).

### 3.5. FHL3 Influences VSMC Proliferation and Migration via MRTFB

**Figure 6:**
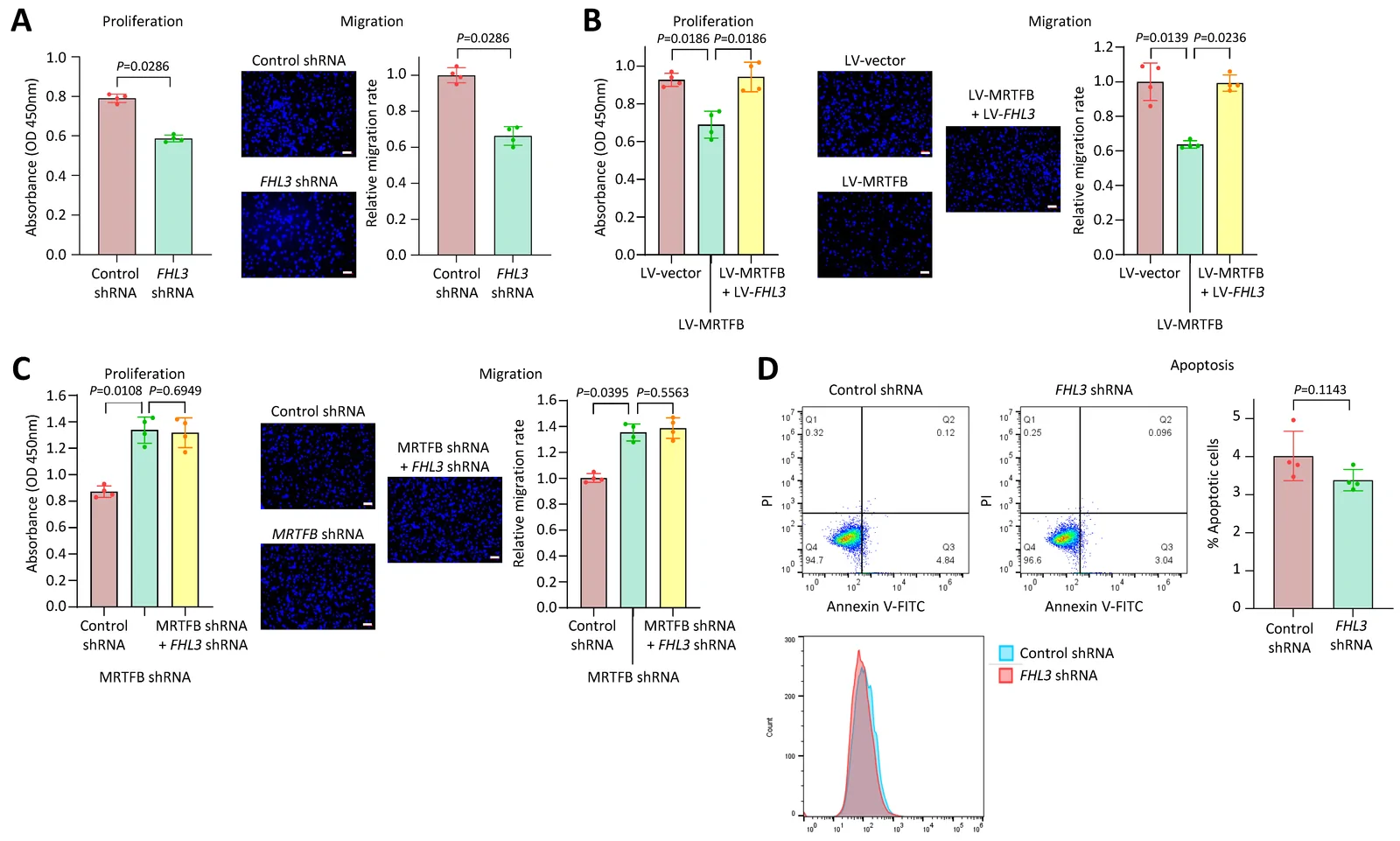
FHL3 promotes VSMC proliferation and migration via MRTFB. (**A**) VSMCs were transduced with either a lentiviral vector to express a non-targeting short hairpin RNA (shRNA) to serve as a control or a lentiviral vector to express a shRNA to target *FHL3* (*FHL3* shRNA), followed by cell proliferation and migration assays. (**B**) VSMCs were transduced with either an empty lentiviral vector (LV-vector) to serve as a control, a lentiviral vector to express *MRTFB* (LV-*MRTFB*), or lentiviral vectors to express *MRTFB* (LV-*MRTFB*) and *FHL3* (LV-*FHL3*), respectively, followed by cell proliferation assay and migration assay. (**C**) VSMCs were transduced with either a lentiviral vector to express a non-targeting short hairpin RNA (shRNA) to serve as a control, a lentiviral vector to express a shRNA to target *MRTFB* (*MRTFB* shRNA), or lentiviral vectors to express shRNAs to target MRTFB (*MRTFB* shRNA) and FHL3 (*FHL3* shRNA), respectively, followed by cell proliferation and migration assays. (**A**–**C**) Cell proliferation was measured by a colorimetric assay with the use of Cell Counting Kit-8, and cell migration was measured by Transwell assay. Representative images of migrated cells stained with Hoechst 33342 are shown. Micrographs were acquired at 10× magnification. Scale bar: 100 µm. (**D**) VSMCs were transduced with either a lentiviral vector to express a non-targeting short hairpin RNA (shRNA) to serve as a control or a lentiviral vector to express a shRNA to target *FHL3* (*FHL3* shRNA), followed by an apoptosis assay with the use of a Dead Cell Apoptosis Kit to stain cells with annexin V-fluorescein isothiocyanate (Annexin V-FITC) and propidium iodide (PI), respectively. Upper: Representative flow cytometry graphs. Bottom: Histogram overlay of Annexin V-FITC fluorescence intensity between control and *FHL3*-knockdown VSMCs, showing minimal signal shift. (**A**–**D**) Column charts show means ± standard deviation. Each plotted data point represents a technical replicate. *p* values from two-sided Mann–Whitney U test or two-sided Kruskal–Wallis test with post hoc Dunn’s test, *n* = 4 technical replicates per condition.

As proliferation and migration are reduced in contractile VSMCs compared to proliferative VSMCs [6] we investigated whether FHL3 could influence VSMC proliferation and migration. We found that *FHL3* knockdown or *MRTFB* overexpression resulted in a decrease in VSMC proliferation and migration and that *FHL3* overexpression counteracted the effect of *MRTFB* overexpression (Figure 6A,B), suggesting that FHL3 influences VSMC proliferation and migration via MRTFB. In support, another assay showed that *MRTFB* knockdown increased VSMC proliferation and migration, while concurrent *FHL3* knockdown did not result in a further change in VSMC proliferation and migration (Figure 6C). Additionally, we investigated whether FHL3 could influence VSMC apoptosis. We observed no significant effect of *FHL3* knockdown on VSMC apoptotic rates (Figure 6D). Consistently, an analysis of reported data [34] showed that the expression of the proliferation marker *Mki67* was reduced in myoblasts with *Fhl3* knockout compared with wildtype myoblasts, whereas the apoptosis marker Casp3 had similar expression levels in myoblasts with *Fhl3* knockout compared with wildtype myoblasts (Supplemental Figure S6).

**Figure 7:**
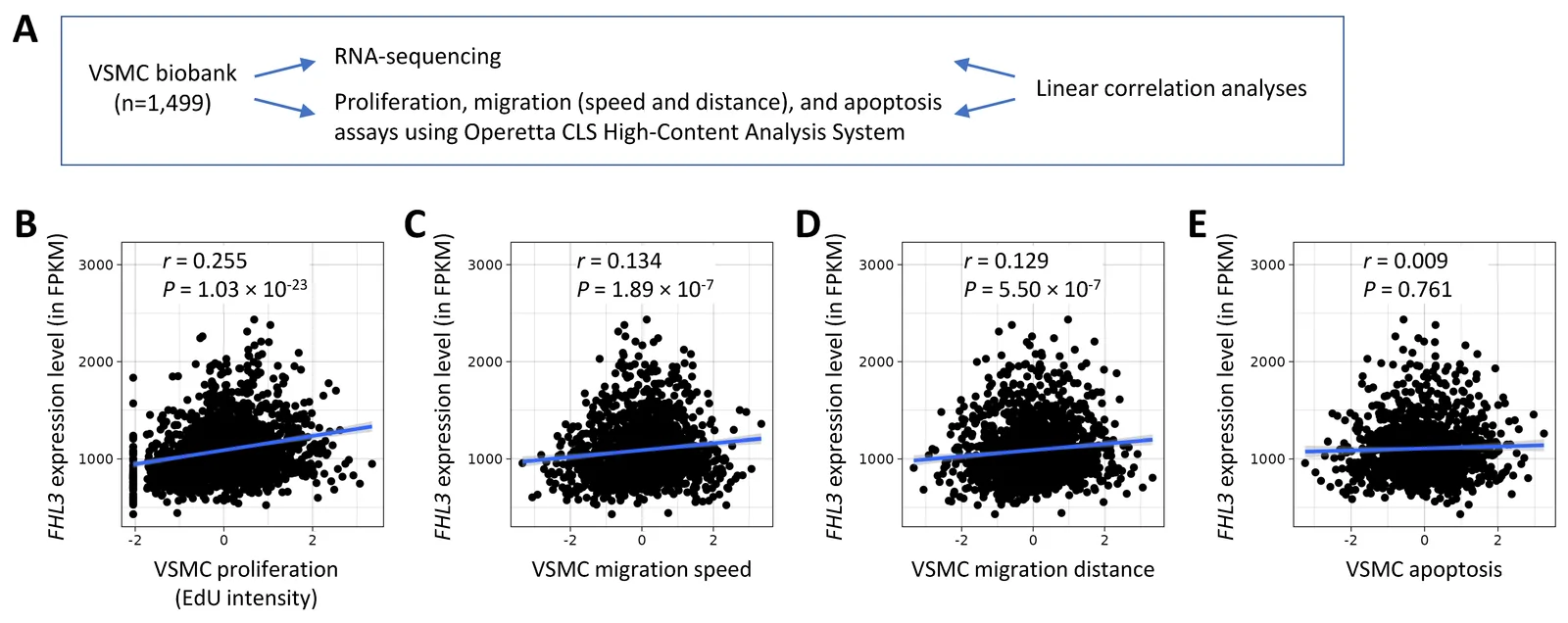
Correlation of FHL3 expression with proliferation and migration of VSMCs. (**A**) A schematic illustration of experimental procedure. (**B**–**E**) Scatter plots showing a correlation of *FHL3* expression levels with proliferation (**B**)**,** migration speed (**C**) and migration distance (**D**), and no correlation with apoptosis (**E**) in VSMCs from different individuals from a VSMC biobank that we recently developed [4]. Proliferation, migration, and apoptosis were determined using an Operetta CLS High-Content Analysis System (PerkinElmer), following staining of the cells with 5-ethynyl-2′-deoxyuridine (EdU, in proliferation assay), CellTracker Green CMFDA (in migration assay), and Hoechst 33342 together with propidium iodide (in apoptosis assay), respectively, as described previously [4]. Each plotted data point represents an independent biological replicate (donor-derived VSMC sample). *p* value from linear correlation analysis; *n* = 1499 biological replicates for both *FHL3* expression and VSMC proliferation/migration data. FPKM: fragments per kilobase of transcript per million mapped reads; *r*: correlation coefficient.

In support, analyses of VSMCs from different individuals (*n* = 1486) showed that there was a correlation of *FHL3* expression levels with VSMC proliferation (Figure 7A) and migration (Figure 7B,C) and no significant correlation with VSMC apoptosis (Figure 7D).

## 4. Discussion

To facilitate pharmaceutical and medical translation from the recent discovery of the many genomic loci associated with CAD, it is important to identify the causal genes at these loci and understand their pathobiological roles. Our recent studies [4,5] identified a number of candidate causal genes at various CAD risk loci that have been previously mapped by GWASs [14,15,16,17], one of which is *FHL3* on chromosome 1p34.3. Our present study reveals that FHL3 modulates VSMC phenotype and behavior, providing insight into the pathobiological role of this CAD gene.

Interestingly, a number of genes located at CAD risk loci identified by GWASs have been shown to influence VSMC phenotypic switching, including *ADAMTS7* [35,36], *FES* [5,37], *FHL5* [38], *HHIPL1* [39] and *TCF21* [40,41]. For example, it has been reported that *TCF21* suppresses the expression of contractile VSMC markers, promotes VSMC phenotypic transition, and thereby reduces CAD risk, indicating a protective role of VSMC phenotypic modulation in atherosclerosis [40,41]. Therefore, it appears that regulating VSMC phenotypic switching is a common mechanism through which many of the genetic loci influence CAD risk. Our findings support *FHL3* as a candidate effector gene through which genetic variation at this CAD locus influences the VSMC phenotype.

VSMC phenotypic modulation is increasingly recognized as a dynamic and context-dependent process during atherosclerosis. While acquisition of a synthetic phenotype contributes to lesion formation, phenotypically modulated VSMCs also migrate into the fibrous cap and contribute to plaque stabilization. Therefore, the biological consequences of modulating the VSMC phenotype are likely to depend on the stage of disease and the vascular microenvironment. In this study, we found that the CAD risk allele is associated with reduced *FHL3* expression, whereas *FHL3* knockdown in cultured VSMCs promotes expression of contractile marker genes and reduces proliferation and migration. These findings are compatible with a hypothesis that the CAD risk allele may increase plaque instability, and therefore CAD risk, due to reduced *FHL3* expression and consequently reduced VSMC content in the plaque cap.

**Figure 8:**
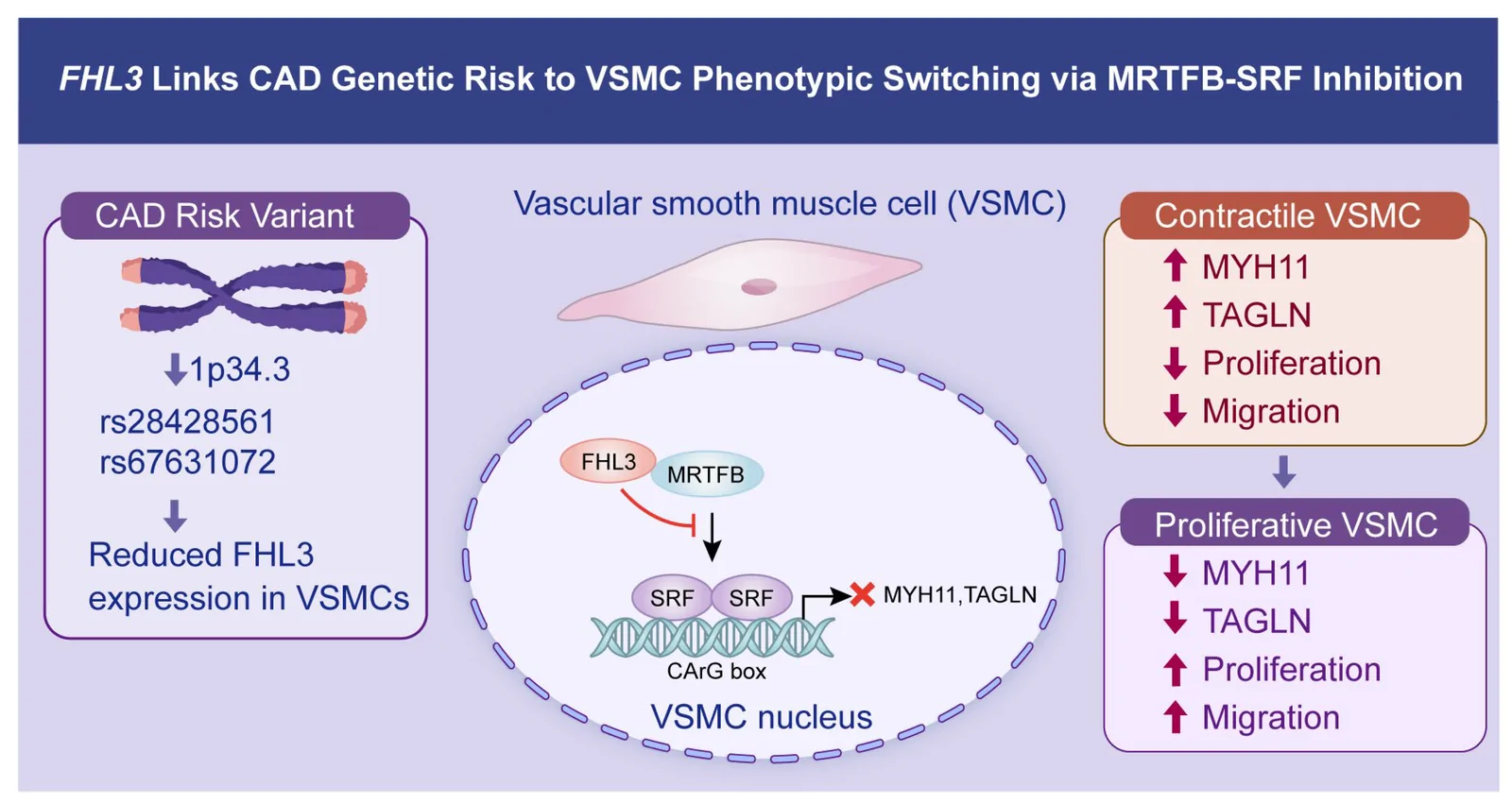
Schematic presentation of the key findings from this study. CAD-associated risk variants reduce expression of *FHL3*, which inhibits the up-regulation of the VSMC contractile genes *MYH11* and *TAGLN* by interfering with the interaction of MRTFB and SRF. Alterations in the VSMC phenotype influence proliferation, migration, and extracellular matrix production, with context-dependent consequences for plaque development and stability. Abbreviations: coronary artery disease, CAD; myocardin-related transcription factor B, MRTFB; myosin heavy chain 11, MYH11; serum response factor, SRF; transgelin, TAGLN.

A hallmark of contractile VSMCs is the expression of certain contractile proteins, with MYH11 considered to be the most specific [8]. It has been established that the transcription factor SRF binds to CArG box elements in promoter regions of these VSMC marker genes, thereby activating their transcription [8]. This is regulated by the co-activators myocardin, MRTFA and MRTFB, which complex with SRF. The results of our present study indicate that FHL3 interferes with this transcription regulatory complex, thereby reducing *MYH11* expression. Specifically, the FHL3 protein is associated with the MRTFB protein and interferes with the interaction between MRTFB and SRF, preventing up-regulation of *MYH11* expression (outlined in Figure 8). Thus, our study suggests an FHL3–MRTFB-SRF axis that regulates the expression of contractile VSMC hallmark proteins.

The four-and-a-half LIM domain (FHL) protein family consists of five members characterized by a unique structural arrangement: a single “half” LIM domain at the N-terminus followed by four complete, tandemly repeated LIM domains [42,43]. These proteins act as molecular scaffolds, co-regulators, or adapters to facilitate protein–protein interactions and are primarily involved in cytoskeletal organization, muscle development, transcription regulation, and signal transduction. While FHL1, FHL2 and FHL3 have been shown to influence skeletal and/or cardiac muscles [42,43,44], FHL2 and FHL5 have been implicated in atherosclerosis [38,45,46]. It is reported that FHL2 can influence VSMC proliferation and migration [47] and that FHL5 promotes vascular remodeling and calcification. In analogy, our current study reveals that FHL3 modulates VSMC phenotype and behavior. Interestingly, both *FHL3* and *FHL5* are candidate causal CAD genes [5,48], residing on chromosome 1p34.3 and 6q16.1, respectively. The findings of these studies add to the growing understanding of the cardiovascular roles of the FHL family.

FHL3 is a cytoskeletal scaffold protein and is also located in the nucleus. To date, most studies of FHL3 have been focused on its functions in skeletal muscles [42,43]. Studies have shown that FHL3 interacts with myoblast determination protein 1 (MYOD) and other transcription factors to modulate the differentiation of myoblasts into mature muscle fibers [44,49], and that *FHL3* overexpression affects muscle glycolytic metabolism with implications in muscle metabolism-related diseases such as type 2 diabetes [34]. Beyond muscle, it is reported that FHL3 either suppresses or promotes tumor progression by modulating epithelial–mesenchymal transition, proliferation, and migration [50]. In several cancers, FHL3 has been reported to modulate signaling pathways including TGF-β, MAPK and NF-κB, whereas in other settings, it has been suggested to function as a tumor suppressor [50]. The finding of our current study that FHL3 modulates the VSMC phenotype expands the understanding of the biological roles of this important protein.

MRTFB is a well-established transcriptional co-activator of SRF and plays a central role in regulating cytoskeletal dynamics, smooth muscle differentiation and myofibroblast activation [51]. Dysregulated MRTFB signaling has been implicated in vascular remodeling, cardiac fibrosis, pulmonary fibrosis, renal fibrosis and several malignancies [51]. Our findings extend this body of work by identifying FHL3 as a possible regulator of MRTFB in human VSMCs.

While our co-immunoprecipitation experiments demonstrate that FHL3 and MRTFB are associated within the same protein complex in VSMCs, they do not distinguish between direct and indirect interactions. Determining whether FHL3 binds directly to MRTFB will require future biochemical studies using purified proteins or other assays capable of resolving direct molecular interactions. Future structural studies, including NMR spectroscopy, X-ray crystallography or cryo-electron microscopy where technically feasible, together with domain-mapping and site-directed mutagenesis, will be required to define the three-dimensional organization and molecular interface of the FHL3-MRTFB complex. Such information may facilitate the development of reagents capable of selectively modulating this interaction.

Although our data support a model in which FHL3 attenuates MRTFB-dependent SRF signaling, the precise molecular mechanism remains to be established. In particular, the present study did not determine whether FHL3 regulates MRTFB abundance, protein stability, post-translational modification, subcellular localization, or chromatin occupancy. Furthermore, we did not investigate potential effects of FHL3 on other SRF co-activators, including MRTFA and myocardin. These important questions warrant future investigation.

In the analyzed arterial tissue, *FHL3* expression was higher in VSMCs, lower in fibroblasts, and lower still in endothelial cells. VSMCs and fibroblasts share several mesenchymal features, including dynamic regulation of the cytoskeleton, extracellular matrix interactions, migration and phenotypic plasticity. The relative enrichment of FHL3 in these cell types may therefore reflect its function as a LIM-domain-containing regulatory protein involved in cytoskeletal and transcriptional processes. However, the present data do not establish the developmental or evolutionary basis of this cell-type-specific expression pattern, which would require comparative lineage and cross-species studies.

One limitation of the present study is that the shRNA-mediated knockdown experiments used a single targeting sequence for *FHL3* and *MRTFB*. Although the conclusions are supported by an independent pooled CRISPR screen, complementary gain-of-function experiments, human genetic analyses, and multiple orthogonal mechanistic approaches, future studies using additional independent shRNAs or CRISPR-mediated gene editing will further strengthen the evidence for target specificity.

Another limitation of the present study is that the mechanistic gain- and loss-of-function experiments were performed in a single commercially available human aortic smooth muscle cell line cultured under in vitro conditions. However, these observations were independently supported by analyses of donor-derived primary human VSMCs from our large VSMC biobank, including genetic, transcriptomic, proliferation, and migration data, providing complementary evidence beyond the mechanistic studies performed in cultured HASMCs. Nevertheless, functional validation in independently isolated primary VSMCs and in vivo or ex vivo vascular models will be important to establish the physiological significance of the FHL3-MRTFB pathway.

Although VSMC proliferation following FHL3 perturbation was assessed using the CCK-8 assay in the present study, the observed association between FHL3 and proliferation is independently supported by EdU-based proliferation measurements in a large VSMC biobank. Likewise, the association between FHL3 and VSMC migration is supported by both Transwell assays following gene perturbation and an independent high-content imaging assay quantifying migration speed and migration distance. Future studies employing direct proliferation assays after FHL3 manipulation and migration assays specifically controlling for proliferation will provide additional validation.

The present study focused on the regulation of the VSMC contractile program, proliferation and migration by the FHL3-MRTFB axis. Whether FHL3 and MRTFB also influence inflammatory activation of VSMCs was not investigated. Future studies examining inflammatory mediators and adhesion molecules under both basal and inflammatory-stimulation conditions will be required to determine whether this pathway also regulates the inflammatory phenotype of VSMCs.

The association of rs67631072 with *FHL3* expression was examined in the coronary artery, aorta and tibial artery because these were the arterial tissues for which suitable eQTL data were available in GTEx. Comparable data were not available for several other vascular beds. Future studies using larger, cell-type-resolved eQTL datasets from additional vascular territories can help determine the generalizability of this regulatory association.

Although our genetic analyses support rs28428561 and rs67631072 as regulatory variants affecting *FHL3* expression, the precise molecular mechanisms remain to be determined. Future studies using allele-specific reporter assays, genome editing, chromatin accessibility analyses and transcription-factor binding assays will be required to identify the regulatory mechanisms through which these variants influence *FHL3* expression. In summary, the findings of this study further the understanding of the molecular mechanisms controlling VSMC phenotypes and provide insight into the pathobiological role of FHL3 encoded by the candidate effector gene *FHL3* at the CAD risk locus on chromosome 1p34.3. CAD risk variant-associated reductions in *FHL3* expression inhibit the phenotypic switching of VSMCs from a contractile to a proliferative/synthetic phenotype, potentially resulting in greater risk of plaque rupture due to a lower proliferative/synthetic VSMC contribution towards plaque stability. This finding highlights the nuanced dichotomy that is VSMC phenotype switching; it contributes to plaque development but also protects against plaque rupture. These findings provide a mechanistic foundation for future studies investigating the therapeutic potential of targeting CAD causal genes and VSMC phenotypic modulation. Whether such approaches can be translated into effective therapies for CAD will require validation in appropriate in vivo models. The opportunities for modulating VSMC behavior to improve cardiovascular outcomes remain relatively underexplored and have recently attracted increasing attention [7].

## Data Availability

The data that support the findings of this study are available from the corresponding author upon reasonable request.

## Supplementary Materials

The following supporting information can be downloaded at: https://www.mdpi.com/article/10.3390/cells15161475/s1, Figure S1: CRISPR screen quality-control metrics; Figure S2: VSMCs in aneurysmal arterial tissues express FHL3; Figure S3: FHL3 expression in various cell types; Figure S4: Genes co-expressed with FHL3 (left) and MRTFB (right); Figure S5: Additional images of images from independent experiments; Figure S6: Reported data of Mki67 and Casp3 expression levels in wildtype (WT) and Fhl3 knockout (KO) mouse myoblasts (C2C12 cells); Table S1: Antibodies used in this study; Table S2: Results of pooled CRISPR-Cas 9 knockout screen; Table S3: Results of Coloc analysis of coronary artery tissues; Table S4: Results of Coloc analysis of aortic tissues; Table S5: Results of Coloc analysis of tibial artery tissues.

## Abbreviations

BSA	bovine serum albumin
CAD	coronary artery disease
Cas9	CRISPR-associated protein 9
cDNA	complementary DNA
CRISPR	clustered regularly interspaced short palindromic repeats
DAPI	4′,6-diamidino-2-phenylindole
DMEM	Dulbecco’s Modified Eagle Medium
EdU	5-ethynyl-2′-deoxyuridine
eQTL	expression quantitative trait locus
FITC	fluorescein isothiocyanate
FHL3	four and a half LIM domains protein 3
FPKM	fragments per kilobase of transcript per million mapped reads
GTEx	Genotype-Tissue Expression
GWAS	genome-wide association study
HASMCs	human aortic smooth muscle cells
MOI	multiplicity of infection
MRTFB	myocardin-related transcription factor-B
MYH11	smooth muscle myosin heavy chain 11
NGS	next-generation sequencing
PBS	phosphate-buffered saline
PCR	polymerase chain reaction
PI	propidium iodide
RNA-seq	RNA sequencing
sgRNA	single-guide RNA
shRNA	short hairpin RNA
SRF	serum response factor
STRING	Search Tool for the Retrieval of Interacting Genes/Proteins
TAGLN	transgelin
UMAP	Uniform Manifold Approximation and Projection
VSMCs	vascular smooth muscle cells
