# Supplementary material for: FHL3 Regulates Vascular Smooth Muscle Cell Phenotypic Switching Through the MRTFB-SRF Signaling Axis
Source: Cells. 2026 Aug 17;15(16):1475. doi: 10.3390/cells15161475 (PMC13510585; doi:10.3390/cells15161475)
Supplement: Supplementary file 1 [file cells-15-01475-s001.zip › Supplementary Figures.pdf]

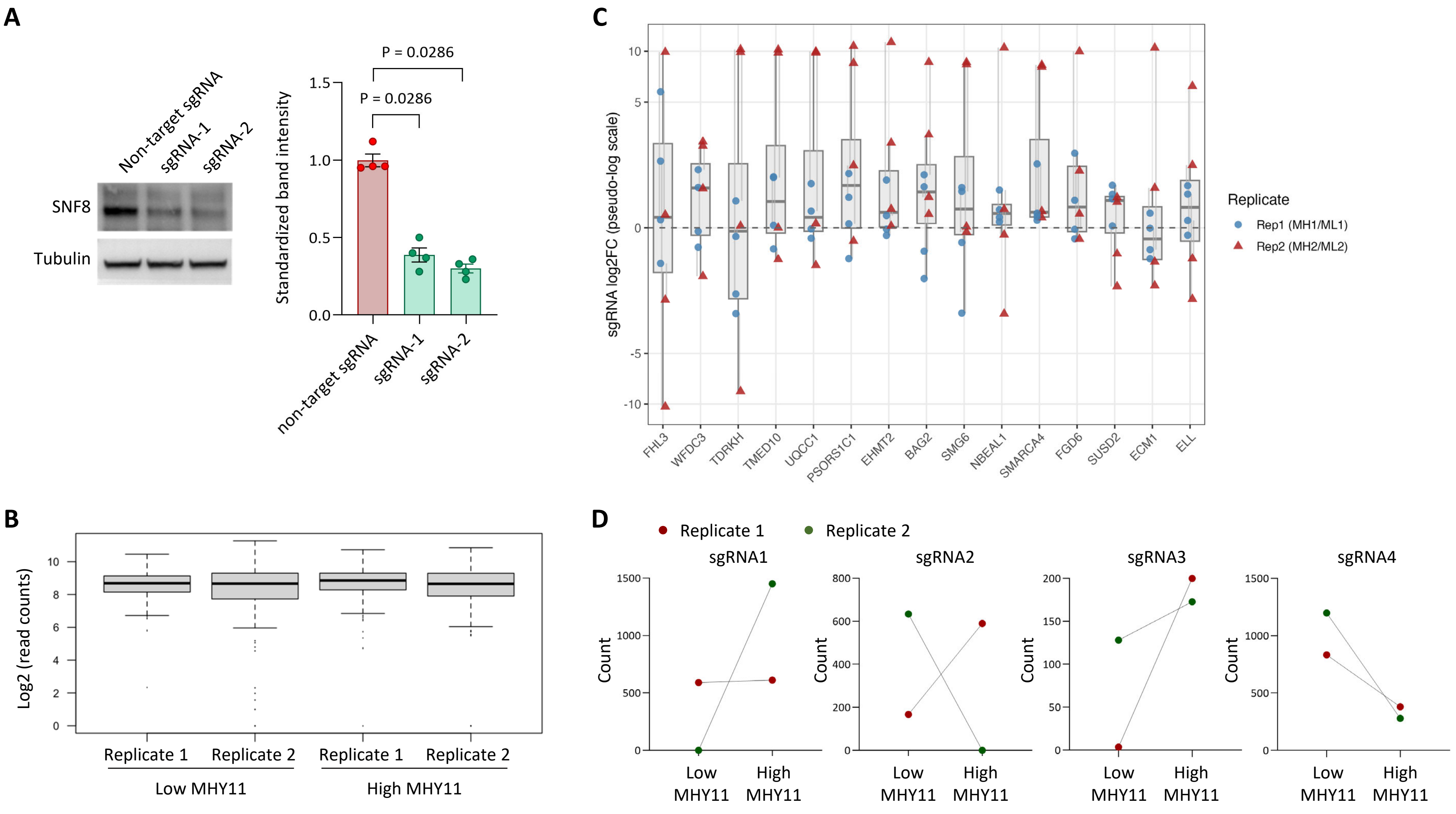

Supplemental Figure S1. CRISPR screen quality-control metrics

A. Efficiency of Cas9-mediated editing. **B**. Distribution of sgRNA counts. **C**. Multi-sgRNA consistency by replicate of the top 15 genes. **D**. Results for the 4 individual *FHL3*-targeting sgRNAs in each technical replicate.

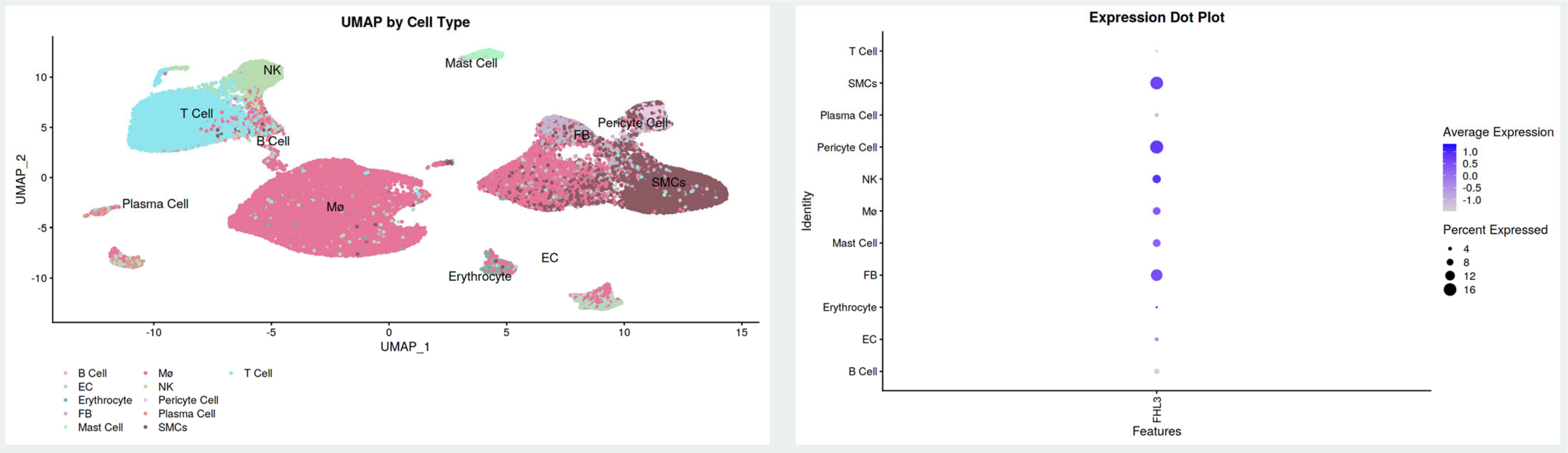

Supplemental Figure S2. VSMCs in aneurysmal arterial tissues express *FHL3*

UMAP (Uniform Manifold Approximation and Projection) visualization (left) and bubble plot (right) of *FHL3* gene expression in various cell types in aneurysmal arterial tissues (data from GSE155468), showing enrichment of *FHL3* in vascular smooth muscle cells (SMC).

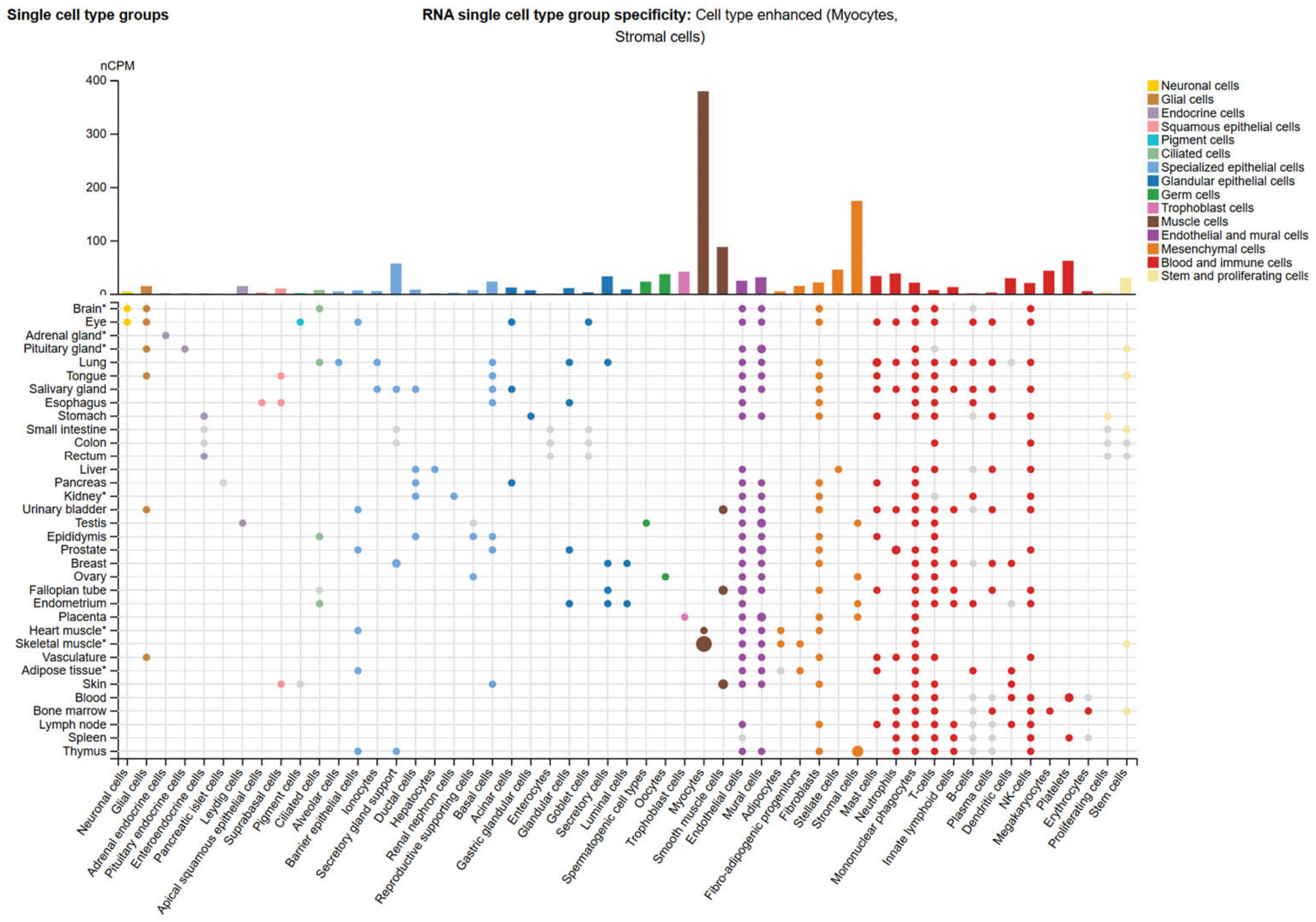

Supplemental Figure S3. ***FHL3*** **expression in various cell types**

Data from Human Protein Atlas (https://www.proteinatlas.org/)

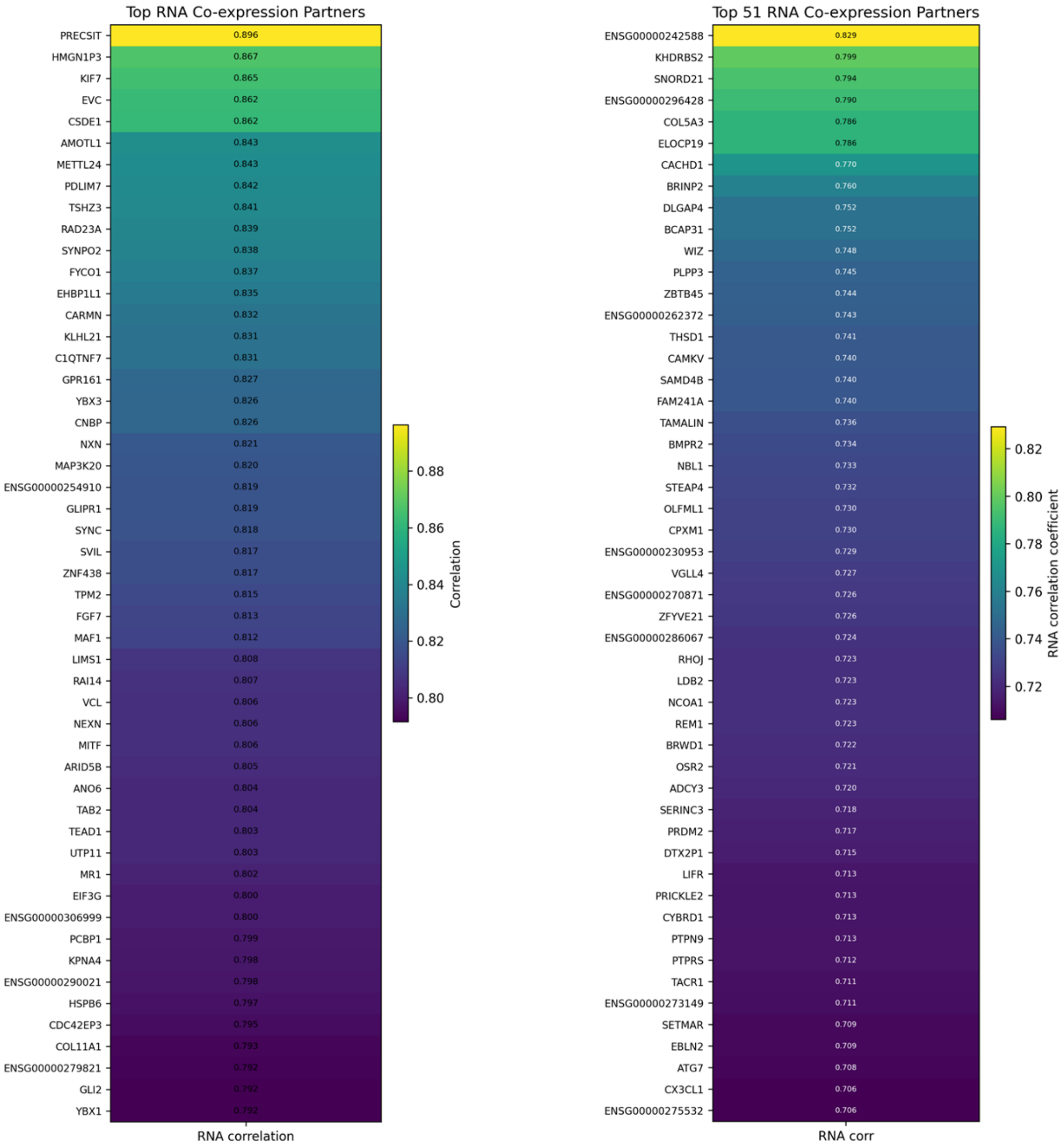

Supplemental Figure S4. Genes co-expressed with *FHL3* (left) and *MRTFB* (right)
Data from GeneCards (https://www.genecards.org/)

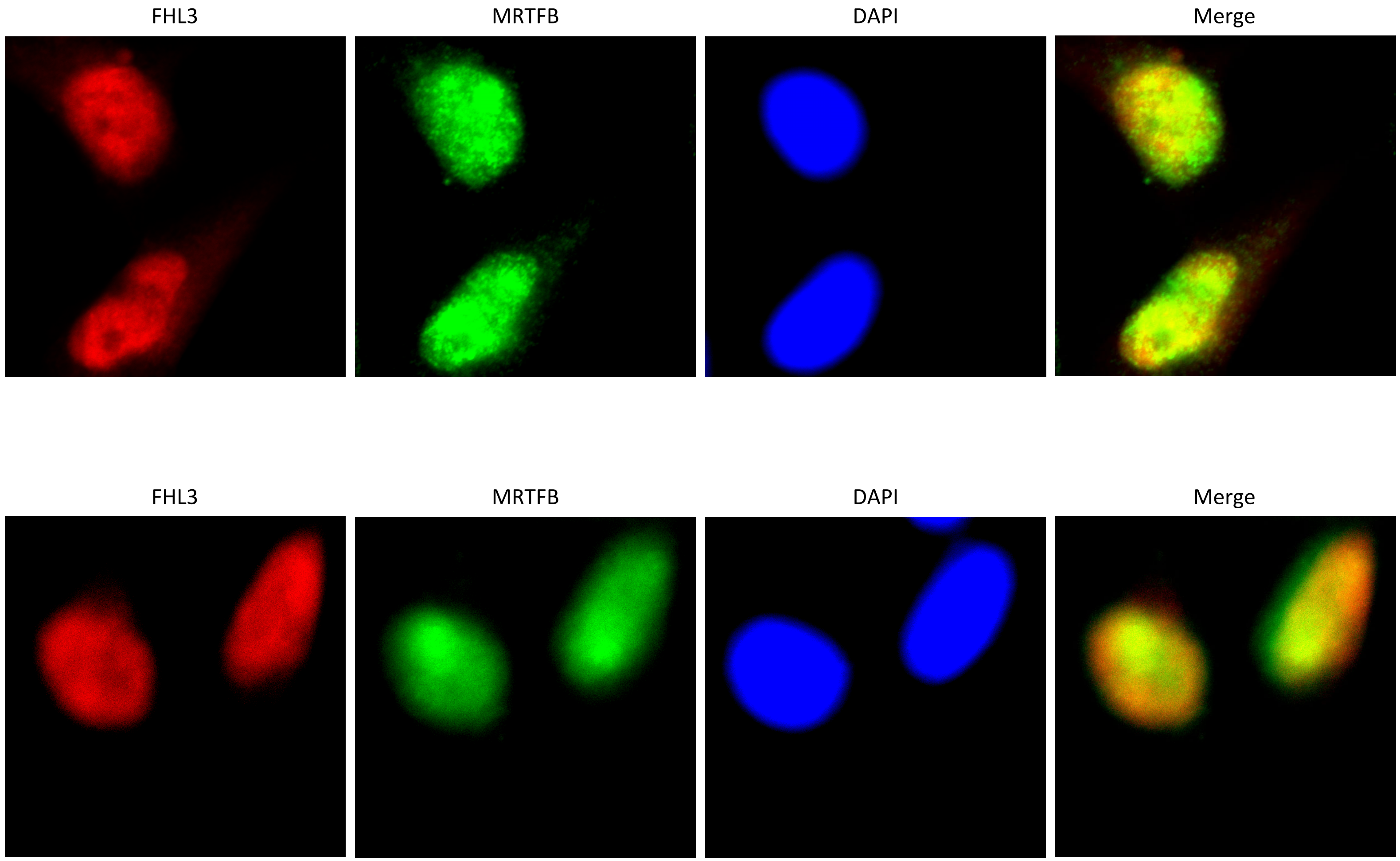

Supplemental Figure S5. Additional images of images from independent experiments

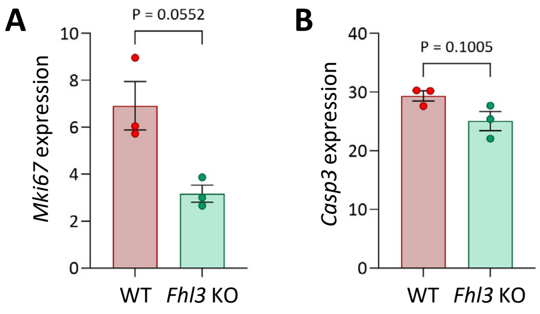

Supplemental Figure S6. Reported data of Mki67 and Casp3 expression levels in wildtype (WT) and *Fhl3* knockout (KO) mouse myoblasts (C2C12 cells)

Data from Gene Expression Omnibus Accession Number GSE213996 by Bai et al (PMID: 36602641).

A. Relative expression levels of *Mki67.* **B**. Relative expression levels of *Casp3.*
